# Inhibitor-Free Variant of the Cheung Regimen: Successful Adjunctive Therapy in Long-Standing Hypochondriasis With Prominent Somatic Features

**DOI:** 10.7759/cureus.105367

**Published:** 2026-03-17

**Authors:** Ngo Cheung

**Affiliations:** 1 Psychiatry, Independent Research, Hong Kong, HKG

**Keywords:** ampa receptors, glutamatergic, nmda receptor antagonist, ocd and related disorders, piracetam

## Abstract

Hypochondriacal obsessive-compulsive disorder (OCD) combines intrusive health anxieties with compulsive reassurance seeking and often resists high-dose selective serotonin reuptake inhibitors and cognitive-behavioural therapy. Interest has shifted toward glutamatergic agents that mimic ketamine's rapid synaptic effects.

A 34-year-old woman endured two years of recurrent fear of serious gastrointestinal and cardiac disease. Escitalopram, later switched to fluoxetine, plus intermittent low-dose atypical antipsychotics and benzodiazepines, produced only partial relief. No psychotic features emerged, yet nightly rumination and repeated medical visits persisted. She agreed to add dextromethorphan 30 mg once daily together with piracetam 600 mg once daily. She declined a cytochrome P450 2D6 (*CYP2D6*)-inhibiting antidepressant, so no pharmacokinetic booster was used. The combination represents a pared-down variant of the Cheung glutamatergic regimen.

Within three weeks, the patient reported the disappearance of nocturnal anxiety, cessation of gastrointestinal preoccupations, and overall mood normalisation; only mild sleep disruption occurred, and no other adverse effects surfaced. Even without *CYP2D6* inhibition, the pairing of low-dose dextromethorphan (N-methyl-D-aspartate antagonism) and piracetam (α-amino-3-hydroxy-5-methyl-4-isoxazolepropionic acid potentiation) may trigger enough neuroplastic change to quiet refractory hypochondriacal OCD. Although confounded by ongoing medications, recent changes to the background regimen, and the absence of *CYP2D6* genotyping or structured OCD ratings, this single observation encourages further controlled study of low-risk glutamatergic augmentation for anxiety-spectrum disorders that defy conventional care.

## Introduction

Illness anxiety disorder (IAD) -- known as hypochondriasis in earlier nosologies -- centres on a persistent fear of serious disease that persists despite normal examinations or mild physical findings. When the preoccupation takes on an intrusive, repetitive quality and is accompanied by compulsive reassurance-seeking rituals, the presentation is widely conceptualised as hypochondriacal obsessive-compulsive disorder (OCD), reflecting shared fronto-striatal circuitry with classic OCD [1]. The condition generates marked distress, limits day-to-day functioning, and fuels repetitive behaviours such as doctor shopping, online searching, or self-examination [2]. The resulting cycle of consultations, investigations, and emergency presentations imposes a substantial burden on healthcare resources and diverts clinical attention from other patients [2].

Standard care mirrors that overlap. Selective serotonin reuptake inhibitors (SSRIs) and cognitive-behavioural therapy (CBT) with exposure and response prevention (ERP) remain first choices for both OCD generally and its hypochondriacal variant, yet a sizeable proportion of patients keep ruminating about illness and remain functionally impaired [3]. Attention has therefore shifted to glutamate, a transmitter repeatedly implicated in OCD: magnetic resonance spectroscopy and cerebrospinal fluid studies reveal hyperglutamatergia, while functional imaging points to cortico-striatal overdrive [4].

Pharmacology has followed suit. Ketamine is a dissociative anaesthetic that acts as an uncompetitive antagonist at N-methyl-D-aspartate (NMDA) receptors, ligand-gated ion channels that normally require both glutamate binding and post-synaptic depolarisation to conduct calcium. A single intravenous dose of ketamine can silence obsessions within hours and, in some cases, for a week [5]. The mechanism is thought to proceed in stages: ketamine preferentially blocks NMDA receptors on gamma-aminobutyric acid (GABAergic) interneurons, transiently disinhibiting glutamatergic pyramidal neurons and unleashing a surge of synaptic glutamate. This glutamate surge activates α-amino-3-hydroxy-5-methyl-4-isoxazolepropionic acid (AMPA) receptors --the principal mediators of fast excitatory transmission in the brain -- whose ion flux triggers the release of brain-derived neurotrophic factor (BDNF). BDNF, in turn, activates the mechanistic target of rapamycin (mTOR) intracellular signalling cascade, a kinase pathway that promotes rapid formation of new dendritic spines, restoring synaptic connectivity in prefrontal-striatal circuits that had become rigid under chronic hyperglutamatergia [5,6]. It is this downstream AMPA-dependent plasticity, rather than NMDA blockade itself, that is believed to produce the lasting therapeutic effect and thus improvement in obsessions.

Translating that hospital-based infusion to routine practice, the oral fixed-dose product dextromethorphan-bupropion (Auvelity®) combines NMDA antagonism with cytochrome P450 2D6 (*CYP2D6*) inhibition -- *CYP2D6* being the principal hepatic enzyme responsible for O-demethylation of dextromethorphan to its less centrally active metabolite dextrorphan -- to keep dextromethorphan active and has shortened onset times in major depressive disorder [7], though its value for OCD-spectrum conditions, including hypochondriacal OCD, is still uncertain.

Building on these insights, Cheung [8] outlined a fully oral, low-cost glutamatergic regimen: dextromethorphan, a morphinan-class antitussive, and NMDA receptor antagonist, for NMDA blockade; a potent *CYP2D6* inhibitor such as fluoxetine or paroxetine to extend dextromethorphan exposure; piracetam, the prototypical racetam-class nootropic, to boost AMPA signalling; and L-glutamine, a conditionally essential amino acid that serves as a precursor for presynaptic glutamate synthesis via the glutaminase pathway and may buffer against excitotoxic damage by maintaining the astrocytic glutamate-glutamine cycle [8]. Early case reports, including hypochondriacal variants, hint at benefit even when the *CYP2D6* inhibitor is omitted, using only dextromethorphan plus piracetam [9], but controlled evidence is not yet available.

The present work describes such a pared-down glutamatergic augmentation in a patient with long-standing hypochondriacal OCD, aiming to add practical detail to this emerging therapeutic avenue.

## Case presentation

Setting and demographics

The patient, a 34-year-old Chinese woman, was seen at a private outpatient psychiatric clinic in Tsim Sha Tsui, Hong Kong, for continuing psychiatric care for hypochondriacal OCD, first diagnosed approximately two years before presentation. She was employed full time and had recently commenced a part-time Master's degree in Psychology. Marital and relationship status were not formally recorded. She had no children. She denied tobacco, alcohol, or recreational drug use at all visits.

Past medical and psychiatric history

She had no documented chronic medical conditions and no prior psychiatric diagnoses before the index episode. There was no reported history of physical or sexual trauma beyond the bereavement described below. No history of substance misuse was recorded.

Family history

Her mother had died of cancer when the patient was approximately 14-15 years old (Form 3 of the Hong Kong secondary school system), an event she identified as the origin of her health vigilance. No family history of psychiatric illness was documented. In March 2025, an unspecified family member required intensive care unit admission, which transiently worsened her anxiety and somatic symptoms.

Investigations

Routine blood investigations (complete blood count, renal function, liver function, and thyroid function), electrocardiography, and upper gastrointestinal evaluation performed by external general practitioners during the two-year illness course were reported to the treating psychiatrist as within normal limits. Original reports were held by the referring providers and were not available for independent review. No new laboratory or imaging investigations were performed at the time of the intervention.

Presenting illness

Her difficulties had begun in late November 2023, approximately two months before her index psychiatric presentation, after a flu-like illness that left her waking abruptly at night with pounding sensations in the back of her head, numb limbs, sweating, and surges of panic. She initially consulted a general practitioner, who prescribed sertraline and an unidentified second medication, and then a private psychiatrist, who prescribed vortioxetine (Brintellix) and alprazolam; neither brief trial provided adequate relief. Those episodes quickly hardened into an all-consuming fear of hidden disease. Minor bodily cues -- most often vague epigastric discomfort or brief palpitations -- were scrutinised and repeatedly checked with physicians or internet searches. The anxiety sat on a long-standing tendency towards health vigilance that dated to her mother's death from cancer during her secondary school years.

Symptom profile

At the time of the index presentation (January 2024), recurring over the subsequent two years, her core symptoms comprised: (a) intrusive, recurrent fears of harbouring a serious gastrointestinal or cardiac illness; (b) heightened daily scanning of bodily sensations, particularly epigastric discomfort, palpitations, and an occipital 'pounding' sensation, intensifying at night; (c) compulsive reassurance seeking through repeated physician visits and internet health searches; and (d) panic-like nocturnal awakenings characterised by autonomic arousal (sweating, tachycardia, and limb numbness). Symptom intensity fluctuated with life stressors such as work deadlines, travel, and a family member's hospitalisation, but the somatic preoccupation never fully remitted on pharmacotherapy alone, though there were periods of substantially reduced anxiety (see below).

Treatment course

Over the subsequent two-year period, she cycled through several guideline treatments (Table 1). Serotonergic trials began with escitalopram 5 mg, increased to 10 mg, and were maintained for approximately 18 months (April 2024-October 2025), then switched to fluoxetine 10 mg in December 2025. Antipsychotic augmentation included risperidone up to 2 mg (January-June 2024), later reduced to 0.5 mg and eventually discontinued by January 2025; aripiprazole 2.5 mg from mid-2024 onward; and night-time quetiapine 12.5-25 mg from late 2024 onward. Benzodiazepines (clonazepam to 1.5 mg/day for approximately two months in April-June 2024, alprazolam 0.25-0.5 mg as required), pregabalin (25-50 mg nightly, approximately one month in July 2024), and lemborexant (5 mg nightly, approximately 10 months from January-November 2024) were added for episodic panic, muscle tension, and fractured sleep. The patient attended one period of counselling (noted July 2024), but no formal CBT with ERP was attempted at any point during the observation period. A persistent 'acid stomach' led to daily rabeprazole 20 mg from December 2025, although investigations were unremarkable. Depressive mood never took hold -- Patient Health Questionnaire-9 (PHQ-9) [10] scores stayed in the 0-4 range, corresponding to minimal depression (PHQ-9 interpretation: 0-4 minimal, 5-9 mild, 10-14 moderate, 15-19 moderately severe, and 20-27 severe) -- but brief spikes of worry (Generalised Anxiety Disorder-7 (GAD-7) [11] scores 0-5; GAD-7 interpretation: 0-4 minimal, 5-9 mild, 10-14 moderate, and 15-21 severe) and recurrent somatic focus lingered, often flaring with work deadlines or breaks in medication while travelling.

**Table 1:** Summary of pharmacological treatments before glutamatergic augmentation (November 2023-December 2025) GP, general practitioner; SSRI, selective serotonin reuptake inhibitor.

Medication	Class	Daily dose	Route/frequency	Approximate duration	Outcome
Sertraline (external GP)	SSRI	Not recorded	Oral; not recorded	Brief trial (late 2023)	Insufficient response; discontinued before index presentation
Vortioxetine (external psychiatrist)	Multimodal antidepressant	Not recorded	Oral; not recorded	Brief trial (late 2023)	Insufficient response; discontinued before index presentation
Escitalopram	SSRI	5→10 mg	Oral; once daily	Apr 2024-Oct 2025 (~18 months)	Partial relief; somatic preoccupation persisted
Fluoxetine	SSRI	10 mg	Oral; once daily	Dec 2025 onward (dispensed)	The patient later reported non-adherence
Risperidone	Atypical antipsychotic	0.5-2 mg	Oral; once daily (night)	Jan-Nov 2024 (~11 months); brief course Mar 2025; re-added Dec 2025	Partial anxiolysis; weight gain at higher doses
Aripiprazole	Atypical antipsychotic	2.5-5 mg	Oral; once daily	Jul 2024 onward	Sleep fragmentation at 5 mg; tolerated at 2.5 mg
Quetiapine	Atypical antipsychotic	12.5-25 mg	Oral; once daily (night)	Brief trial Jan 2024; Sep 2024 onward	Sleep consolidation
Clonazepam	Benzodiazepine	1.5 mg (0.5 mg TDS)	Oral; three times daily	Apr-Jun 2024 (~2 months)	Anxiolysis; tapered and discontinued
Alprazolam	Benzodiazepine	0.25-0.5 mg	Oral; once daily/PRN	Apr 2024 onward	Partial anxiolysis; continued as needed
Pregabalin	Gabapentinoid	25-50 mg	Oral; once daily (night)	Jul 2024 (~1 month)	Brief trial; discontinued
Lemborexant	Dual orexin receptor antagonist	5 mg	Oral; once daily (night)	Jan-Nov 2024 (~10 months)	Improved sleep onset; later stopped per patient preference
Rabeprazole	Proton pump inhibitor	20 mg	Oral; once daily	Dec 2025 onward	Epigastric symptom relief

All medications administered orally. Side effects were not systematically recorded beyond weight gain with risperidone (noted July 2024) and sleep fragmentation with aripiprazole 5 mg (noted August 2024).

Pre-intervention status and recent changes

By mid-2025, while maintained on escitalopram 10 mg, aripiprazole 2.5 mg, quetiapine 12.5 mg, and alprazolam 0.5 mg as needed, the patient reported substantially reduced anxiety (PHQ-9 0, GAD-7 0 in October 2025), was engaged in full-time work and postgraduate study, and had been off risperidone for several months. However, in November 2025, she travelled and inadvertently omitted all her medications for an unspecified period. A symptom flare followed: at the 13 December 2025 visit, she reported recurrence of gastrointestinal discomfort, increased worry (GAD-7 4, mild anxiety range), and work-related stress. Escitalopram was switched to fluoxetine 10 mg daily at that visit; three days later (16 December 2025), risperidone 0.5 mg was re-added along with rabeprazole 20 mg for epigastric complaints. She remained symptomatic over the following month on this revised regimen.

Physical examination and mental status

Physical examination on the day of the intervention was unremarkable: vital signs were within normal limits, and no focal neurological deficits were elicited. Mental status examination revealed an alert, cooperative woman with euthymic mood, mildly anxious affect, no perceptual disturbances, no delusions, and intact cognition. No psychotic features were present at any point during her treatment course. No formal, structured psychometric instruments beyond the PHQ-9 and GAD-7 were administered at this visit.

Intervention

On 10 January 2026 (designated Day 1), she agreed to try a simplified form of the Cheung glutamatergic regimen -- dextromethorphan 30 mg each morning (two 15 mg tablets) plus piracetam 600 mg once daily at night -- added to her current background medications: risperidone 0.5 mg once daily (on board since 16 December 2025), aripiprazole 2.5 mg once daily, quetiapine 12.5 mg once nightly, alprazolam 0.25 mg as needed, and rabeprazole 20 mg once daily. L-glutamine was not included in this regimen. Although fluoxetine 10 mg was dispensed, the patient elected not to take it, thereby omitting the *CYP2D6*-inhibiting component of the full Cheung regimen [8]. No psychotherapy referral was made at any point during the observation period.

Outcome

Review on 31 January 2026 (Day 21) revealed a striking change. She described waking 'clear-headed', free of abdominal discomfort, and no longer compelled to scan her body or consult Dr. Google. All previously reported somatic preoccupations -- epigastric discomfort, palpitations, nocturnal occipital sensations, and panic-like awakenings -- had ceased. Residual anxiety had vanished; sleep, though lighter -- she described more frequent brief nocturnal awakenings but no difficulty returning to sleep and no daytime somnolence - was satisfactory, and no dissociative feelings or rebound panic had appeared. She confirmed full adherence to dextromethorphan and piracetam, continued to decline fluoxetine, and was counselled to try morning dosing of the full regimen while monitoring sleep. No formal psychometric reassessment (PHQ-9, GAD-7, or Yale-Brown Obsessive Compulsive Scale (Y-BOCS)) was performed at this visit. Longer-term follow-up data are not yet available.

In sum, a three-week course of dextromethorphan paired only with piracetam was temporally associated with rapid remission of health-centred obsessions and anxiety in a patient who had derived partial and fluctuating benefit from conventional multimodal therapy. It should be noted that recent changes to the background regimen -- the switch from escitalopram to fluoxetine, re-addition of risperidone, and resumption of medications after a non-adherence gap -- may have contributed to the observed improvement, a point developed further in the Discussion. The response hints that even without formal *CYP2D6* inhibition, a brief NMDA blockade coupled with modest AMPA potentiation may be enough to reset refractory somatic preoccupation in selected cases.

Written informed consent for publication of this case report was obtained from the patient. No identifying information beyond what is necessary for scholarly reporting is disclosed.

## Discussion

Adding low-dose dextromethorphan and piracetam to this patient's recently adjusted regimen was associated with the resolution of two years of recurrent illness-focused obsessions in less than a month, even though no *CYP2D6* inhibitor was used to lengthen dextromethorphan exposure. The result suggests that the full four-drug Cheung stack may not be obligatory in every case; a leaner version that supplies only a brief NMDA blockade (dextromethorphan) plus continuous AMPA potentiation (piracetam) may sometimes contribute to tipping maladaptive circuits back towards normal function.

Dextromethorphan is a morphinan derivative available over the counter as an antitussive. At the doses used for neuropsychiatric purposes, it functions as an uncompetitive NMDA receptor antagonist and a sigma-1 receptor agonist, sharing key pharmacological actions with the dissociative anaesthetic ketamine [6]. Piracetam, the prototypical member of the racetam class of nootropics, lacks the sedative and dependence properties of conventional anxiolytics; its principal documented action is positive allosteric modulation of AMPA-type glutamate receptors [12,13].

AMPA receptors are ligand-gated cation channels that mediate the majority of fast excitatory synaptic transmission in the central nervous system. Glutamate binding opens the channel, permitting sodium influx and depolarising the post-synaptic membrane. Under normal conditions, AMPA receptor activation is essential for learning and synaptic plasticity; in OCD spectrum disorders, however, tonic hyperglutamatergia is thought to desensitise these receptors and impair the activity-dependent plasticity needed to extinguish maladaptive circuit patterns [4].

**Figure 1:**
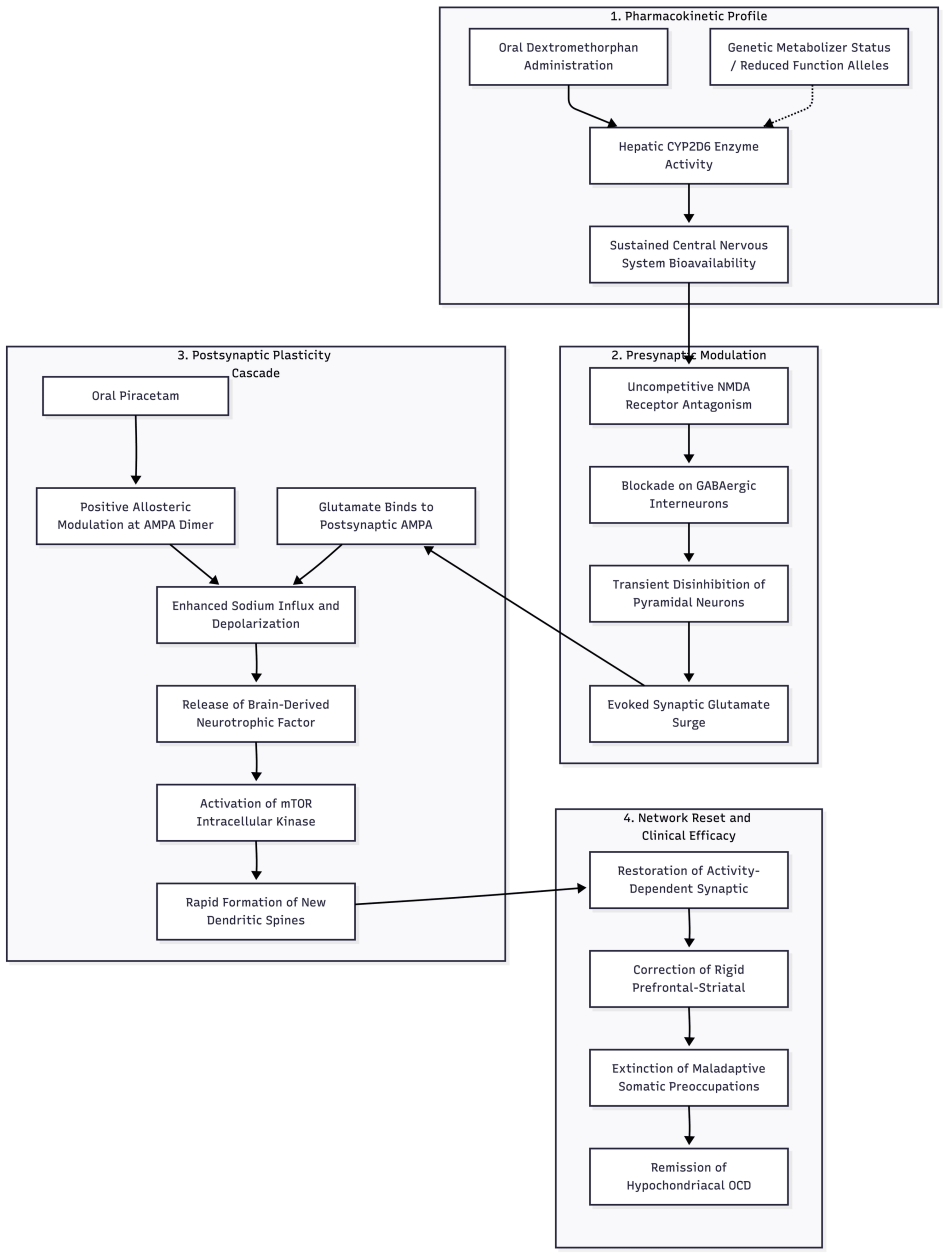
Proposed mechanism of dextromethorphan-piracetam augmentation in hypochondriacal OCD Proposed mechanism of dextromethorphan-piracetam augmentation in hypochondriacal OCD. The pathway proceeds through six stages: (1) dextromethorphan blocks NMDA receptors on GABAergic interneurons, leading to disinhibition of glutamatergic pyramidal neurons and a resultant transient surge in synaptic glutamate; (2) disinhibition of glutamatergic pyramidal neurons produces a transient glutamate surge; (3) glutamate activates post-synaptic AMPA receptors, an effect amplified by piracetam's positive allosteric modulation at the receptor dimer interface; (4) enhanced AMPA throughput triggers BDNF release; (5) BDNF activates the mTOR signalling cascade; (6) mTOR-dependent dendritic spine formation restores synaptic connectivity in prefrontal-striatal circuits, reducing obsessive-compulsive symptoms. Image credit: Ngo Cheung

Basic work offers a plausible mechanism (summarised in Figure 1). In mice, the antidepressant-like action of dextromethorphan disappears when AMPA receptors are blocked with 2,3-dioxo-6-nitro-1,2,3,4-tetrahydrobenzo(f)quinoxaline-7-sulfonamide, a selective AMPA receptor antagonist, confirming that downstream AMPA throughput -- rather than NMDA antagonism per se -- drives behavioural change [14]. Piracetam binds to low-affinity sites along the AMPA receptor dimer interface, reduces desensitisation, and boosts current flow [12,13]. Coupling that action to the transient glutamate surge triggered by dextromethorphan could therefore amplify the BDNF- and mTOR-dependent spine formation that follows ketamine infusions and, by extension, relieve obsessive rumination rooted in rigid prefrontal-striatal loops [6]. In the full Cheung regimen, supplemental L-glutamine serves as a precursor for presynaptic glutamate synthesis via the glutaminase pathway and may buffer against excitotoxic damage by maintaining the astrocytic glutamate-glutamine cycle [8]; it was not included in the present case.

The present observation parallels a prior report by the same author [9], in which a male patient with obsessive ruminations experienced remission on dextromethorphan and piracetam with only minimal *CYP2D6* inhibition from a low-dose SSRI. In both cases, improvement emerged within the first three weeks and was accompanied by mild sleep disruption without dissociative or other serious adverse effects. The consistency of the timeframe and symptom profile across two independent cases -- one with partial *CYP2D6* inhibition and one without -- supports the hypothesis that piracetam-mediated AMPA potentiation may partially compensate for limited dextromethorphan bioavailability, although neither observation permits causal inference.

Pharmacokinetics ordinarily argue against using dextromethorphan without metabolic protection: in extensive metabolisers, bioavailability is near 2% and the parent drug's half-life averages 4 hours [15]. Poor metabolisers or subjects given a *CYP2D6* inhibitor can show 20- to 25-fold higher exposure [16,17]. *CYP2D6* is encoded by a highly polymorphic gene: over 100 allelic variants have been catalogued, ranging from fully functional alleles (e.g., 1 and 2) through reduced functional alleles (e.g., 9, 10, and 41) to non-functional alleles (e.g., 3, 4, and 5) [17]. Individuals carrying two reduced or non-functional alleles are classified as 'poor metabolisers' (approximately 5%-10% of European descent populations and 1%-2% of East Asian descent populations) and exhibit markedly slowed dextromethorphan clearance. Those with one reduced function allele are classified as 'intermediate metabolisers' and may also show clinically significant increases in parent drug exposure [15,17]. In the present patient, who is of East Asian descent, the CYP2D610 allele -- the most prevalent reduced function variant in this population -- could plausibly confer intermediate metaboliser status and partially explain the therapeutic response at 30 mg daily without exogenous enzyme inhibition, although genotyping was not performed. Yet the present case, treated with only 30 mg once daily, reached apparent full remission. One possibility is that piracetam lowered the threshold of glutamatergic signalling required for plastic change, compensating for the drug's rapid clearance.

There are real benefits to omitting the inhibitor. It lowers the risk of dissociation -- a state of altered awareness characterised by detachment from one's surroundings, body, or sense of identity, ranging from mild depersonalisation to frank derealisation [2] -- and of serotonin syndrome, a potentially life-threatening drug interaction syndrome characterised by altered mental status, autonomic instability (hyperthermia, tachycardia, and diaphoresis), and neuromuscular hyperactivity (clonus and rigidity), typically precipitated by combining serotonergic agents [18]. Omission also reduces drug-drug interactions with β-blockers or tamoxifen [8]. It also makes it easier to fill the prescription and may help people stick to it better, as shown by the patient who refused fluoxetine but kept taking the core agents. The trade-off is a shorter dwell time for dextromethorphan and possibly less long-lasting benefit; the mild sleep disturbance she reported could mean that the drug is wearing off in the latter part of the night, a pattern that has been observed in the prior split-dose case report, where once-daily dextromethorphan was associated with symptom return in the evening hours and twice-daily dosing appeared to provide more sustained coverage [9].

Indications, contraindications, and adverse effects

The dextromethorphan-piracetam combination described here is experimental and carries no approved psychiatric indication. Off-label use should be considered only in adults with treatment-resistant OCD spectrum symptoms who have failed adequate SSRI and CBT trials; paediatric safety data are absent. Dextromethorphan is contraindicated in patients receiving monoamine oxidase inhibitors (risk of serotonin syndrome) and warrants caution in hepatic impairment, known *CYP2D6* poor metaboliser status (risk of supratherapeutic exposure), and a history of substance misuse. Piracetam is contraindicated in severe renal impairment and Huntington's disease. Anticipated adverse effects of the combination include mild sleep disruption (as observed here), potential dissociation or derealisation at higher dextromethorphan doses, gastrointestinal upset, and headache. Serotonin syndrome remains a theoretical concern when dextromethorphan is co-administered with serotonergic antidepressants, even at low doses [8,18]. In the present case, only mild sleep disruption was observed over three weeks.

Important confounders

Several factors beyond dextromethorphan and piracetam may have contributed to the observed improvement and must be explicitly acknowledged. First, the symptom flare that immediately preceded the intervention was likely precipitated by medication non-adherence during travel in November 2025 rather than by intrinsic treatment resistance; the subsequent improvement may therefore partly reflect pharmacological re-stabilisation as resumed medications reached steady state. Second, the background regimen was not static in the weeks before the intervention: escitalopram was switched to fluoxetine, risperidone 0.5 mg was reintroduced (having been off the regimen since early 2025), and rabeprazole was started, all within the four weeks before dextromethorphan and piracetam were added. Risperidone, a dopamine D₂ and serotonin 5-HT₂A antagonist with demonstrated anxiolytic properties, may itself have contributed to the improvement. Third, although the patient declined fluoxetine, both aripiprazole and quetiapine are *CYP2D6* substrates that could conceivably exert minor competitive inhibition at the enzyme, modestly slowing dextromethorphan metabolism beyond what would occur in a truly uninhibited state. Fourth, the longitudinal record documents extended periods of relative stability on conventional treatment alone (PHQ-9 0, GAD-7 0 in mid-2025), suggesting a waxing and waning course; a three-week observation window cannot reliably distinguish a pharmacological effect from natural fluctuation.

While SSRI doses were not maximal, the prolonged incomplete remission supports pharmacoresistance in this context; however, the temporal proximity of risperidone re-addition and other regimen changes may account for part of the symptom resolution, limiting causal inference to the glutamatergic agents. 

Although SSRI doses were sub-maximal, the prolonged partial response supports a degree of pharmacoresistance; however, risperidone's re-addition and other adjustments preclude definitive attribution of remission to the inhibitor-free regimen alone. 

Strengths

Notwithstanding the above, the observation has several strengths: a detailed longitudinal record spanning two years of conventional treatment provides robust documentation of a partial, fluctuating treatment response against which the rapid, comprehensive improvement after Day 1 can be judged; the temporal association between dextromethorphan-piracetam initiation and symptom resolution is clinically striking in both timing and completeness; and the regimen was well tolerated with no serious adverse effects.

## Conclusions

Interpretation is constrained by several factors: (a) multiple concurrent medications, including recently re-introduced risperidone, may have influenced the improvement, precluding causal attribution to dextromethorphan and piracetam; (b) the symptom flare that prompted the intervention followed a period of medication non-adherence during travel, and the response may partly reflect pharmacological re-stabilisation; (c) symptom assessments relied on clinical interview rather than validated OCD-specific instruments such as the Y-BOCS or the Health Anxiety Inventory; (d) the follow-up period was limited to three weeks in a condition known for waxing and waning severity; (e) the single-subject design prohibits causal or generalisable inference; and (f) *CYP2D6* genotyping was not performed, leaving the pharmacokinetic basis of the response unknown.

The use of PHQ-9 and GAD-7, while practical for screening comorbid symptoms, lacks specificity for hypochondriacal OCD; validated instruments such as the Y-BOCS should be prioritised in subsequent studies. In this retrospective case observation from a private practice, PHQ-9 and GAD-7 were employed for routine screening of overlapping symptoms rather than targeted OCD/IAD assessment; future prospective studies should incorporate specialised tools such as the Y-BOCS or Health Anxiety Inventory. Nonetheless, the temporal association between glutamatergic augmentation and rapid, comprehensive symptom resolution, coupled with converging preclinical evidence and a parallel case report, warrants formal trials of dextromethorphan-piracetam combinations, both with and without *CYP2D6* inhibition, in hypochondriacal OCD and associated disorders. Such trials should incorporate standardised OCD and health-anxiety outcome measures (e.g., Y-BOCS and Health Anxiety Inventory), *CYP2D6* genotyping, a stable-baseline run-in period of adequate duration, and follow-up of at least 12 weeks to distinguish sustained response from transient fluctuation.

## References

[1] Starcevic V (2013). Hypochondriasis and health anxiety: conceptual challenges. Br J Psychiatry.

[2] American Psychiatric Association (2013). Diagnostic and Statistical Manual of Mental Disorders, Fifth Edition.

[3] Greeven A, van Balkom AJ, van der Leeden R, Merkelbach JW, van den Heuvel OA, Spinhoven P (2009). Cognitive behavioral therapy versus paroxetine in the treatment of hypochondriasis: an 18-month naturalistic follow-up. J Behav Ther Exp Psychiatry.

[4] Pittenger C (2015). Glutamatergic agents for OCD and related disorders. Curr Treat Options Psychiatry.

[5] Rodriguez CI, Kegeles LS, Levinson A (2013). Randomized controlled crossover trial of ketamine in obsessive-compulsive disorder: proof-of-concept. Neuropsychopharmacology.

[6] Stahl SM (2019). Dextromethorphan/bupropion: a novel oral NMDA (N-methyl-d-aspartate) receptor antagonist with multimodal activity. CNS Spectr.

[7] Tabuteau H, Jones A, Anderson A, Jacobson M, Iosifescu DV (2022). Effect of AXS-05 (dextromethorphan-bupropion) in major depressive disorder: a randomized double-blind controlled trial. Am J Psychiatry.

[8] Cheung N (2026). Cheung N: DXM, CYP2D6-inhibiting antidepressants, piracetam, and glutamine: proposing a ketamine-class antidepressant regimen with existing drugs. https://www.preprints.org/manuscript/202511.1815.

[9] Cheung N (2025). Remission of obsessive ruminations using a low-intensity glutamatergic stack: a retrospective case observation of dextromethorphan and piracetam with minimal CYP2D6 inhibition. Zenodo.

[10] Kroenke K, Spitzer RL, Williams JB (2001). The PHQ-9: validity of a brief depression severity measure. J Gen Intern Med.

[11] Spitzer RL, Kroenke K, Williams JB, Löwe B (2006). A brief measure for assessing generalized anxiety disorder: the GAD-7. Arch Intern Med.

[12] Ahmed AH, Oswald RE (2010). Piracetam defines a new binding site for allosteric modulators of alpha-amino-3-hydroxy-5-methyl-4-isoxazole-propionic acid (AMPA) receptors. J Med Chem.

[13] Copani A, Genazzani AA, Aleppo G, Casabona G, Canonico PL, Scapagnini U, Nicoletti F (1992). Nootropic drugs positively modulate alpha-amino-3-hydroxy-5-methyl-4-isoxazolepropionic acid-sensitive glutamate receptors in neuronal cultures. J Neurochem.

[14] Nguyen L, Matsumoto RR (2015). Involvement of AMPA receptors in the antidepressant-like effects of dextromethorphan in mice. Behav Brain Res.

[15] Schmid B, Bircher J, Preisig R, Küpfer A (1985). Polymorphic dextromethorphan metabolism: co-segregation of oxidative O-demethylation with debrisoquin hydroxylation. Clin Pharmacol Ther.

[16] Kronbach T, Mathys D, Umeno M, Gonzalez FJ, Meyer UA (1989). Oxidation of midazolam and triazolam by human liver cytochrome P450IIIA4. Mol Pharmacol.

[17] Grzegorzewski J, Brandhorst J, König M (2022). Physiologically based pharmacokinetic (PBPK) modeling of the role of CYP2D6 polymorphism for metabolic phenotyping with dextromethorphan. Front Pharmacol.

[18] Boyer EW, Shannon M (2005). The serotonin syndrome. N Engl J Med.

